# A Cell’s Fate: An Overview of the Molecular Biology and Genetics of Apoptosis

**DOI:** 10.3390/ijms20174133

**Published:** 2019-08-24

**Authors:** Giovanna C. Cavalcante, Ana Paula Schaan, Gleyce Fonseca Cabral, Mayara Natália Santana-da-Silva, Pablo Pinto, Amanda F. Vidal, Ândrea Ribeiro-dos-Santos

**Affiliations:** 1 Laboratório de Genética Humana e Médica, Universidade Federal do Pará, Belém 66075-110, PA, Brazil; 2 Núcleo de Pesquisas em Oncologia, Universidade Federal do Pará, Belém 66073-005, PA, Brazil

**Keywords:** regulated cell death, apoptosis, mitochondria, miRNAs, genetics

## Abstract

Apoptosis is one of the main types of regulated cell death, a complex process that can be triggered by external or internal stimuli, which activate the extrinsic or the intrinsic pathway, respectively. Among various factors involved in apoptosis, several genes and their interactive networks are crucial regulators of the outcomes of each apoptotic phase. Furthermore, mitochondria are key players in determining the way by which cells will react to internal stress stimuli, thus being the main contributor of the intrinsic pathway, in addition to providing energy for the whole process. Other factors that have been reported as important players of this intricate molecular network are miRNAs, which regulate the genes involved in the apoptotic process. Imbalance in any of these mechanisms can lead to the development of several illnesses, hence, an overall understanding of these processes is essential for the comprehension of such situations. Although apoptosis has been widely studied, the current literature lacks an updated and more general overview on this subject. Therefore, here, we review and discuss the mechanisms of apoptosis, highlighting the roles of genes, miRNAs, and mitochondria involved in this type of cell death.

## 1. Introduction

The mechanisms underlying cell death and survival have a great impact on maintaining cellular balance, such that their deregulation may lead to the development of various diseases, such as multiple types of cancer and neurodegenerative disorders [1,2]. The classifications about cell death modalities depend mainly on morphological and structural details of individual tissues and cells [3]. Among the different types of cell death, apoptosis stands out as one of the most widely studied in the past years. Still, only a few articles provide a general and descriptive overview of apoptosis. 

**Figure 1:**
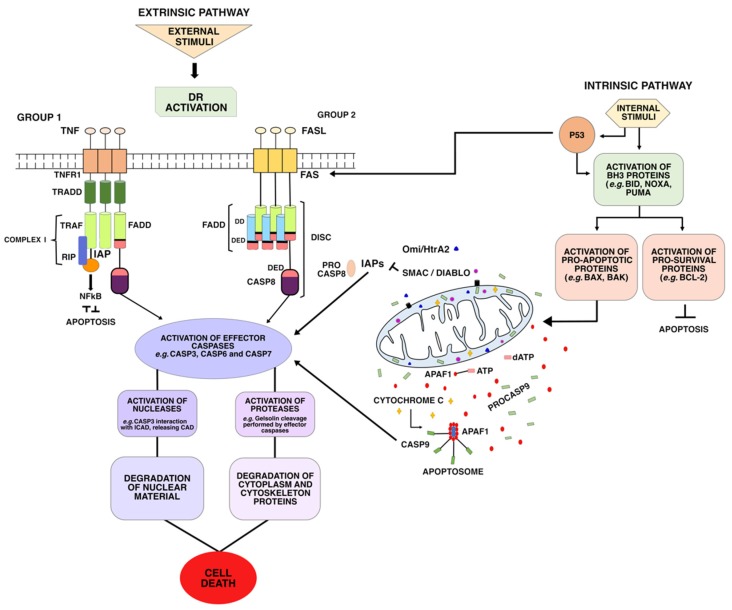
General scheme of the process of apoptotic cell death. This phenomenon may occur via the extrinsic or intrinsic pathway, converging in the executing phase.

Apoptosis is a normal mechanism that can occur at any stage of the individual’s development or upon cell damage, and is marked by the following characteristics: Protein cleavage (occurring mainly by the activation of cysteine proteases known as caspases), nuclear DNA breakdown, and apoptotic cell recognition by phagocytic cells [4]. These processes are responses to internal (intrinsic or mitochondrial) or external (extrinsic pathway or death receptors, DR) stimuli to the cell, converging in the final stage, known as the apoptotic execution phase (Figure 1). 

There are many intricate aspects to both apoptotic pathways and the numerous molecules involved in their mechanisms. The study of the apoptotic pathway has shown to be a great approach in the search for new anticancer therapy, since promising compounds that trigger apoptosis are often non-toxic to healthy cells [5].

In this review, we begin by presenting a general overview of intrinsic and extrinsic pathways and the executioner phase. In more detail, we discuss the roles of major genes involved in apoptosis and highlight the importance of considering epigenetic mechanisms as regulators of the apoptotic machinery.

## 2. Intrinsic or Mitochondrial Pathway

The intrinsic pathway of apoptosis, also known as the mitochondrial pathway, originates due to internal cellular stress [2]. These internal stimuli, such as DNA damage or endoplasmic reticulum stress, induce the activation of BH3-only (BCL-2 homology 3) proteins, which include BID, BIM, BAD, BIK, BMF, Noxa, PUMA, and HRK. BH3 proteins interact and activate BH3 pro-survival or pro-apoptotic proteins, which may lead to the interruption of apoptotic cell death or to the continuation of this process, respectively [6].

In the first scenario, pro-survival proteins (BCL-2, BCL-X, BCL-w, MCL-1, or BFL-1/A1) prevail, interrupting cell death mechanisms at that given point [7]. In the second scenario, the activation of pro-apoptotic proteins BAX (BCL-2-associated X protein) or BAK (BCL-2 antagonist or killer) lead to mitochondrial outer membrane permeabilization (MOMP), considered a point of no return due to the release of proteins involved in the activation of caspases in the cytoplasm, accompanied by the gradual acidification of the environment around the mitochondria [8,9].

Among proteins released through MOMP, there are cytochrome-c (Cyt-c), involved in the formation of apoptosomes, and the pro-apoptotic proteins Smac/DIABLO and Omi/HtrA2, which interact with inhibitory proteins to activate procaspases such as Procaspase-3 (ProC3) and Procaspase-7 (ProC7) [10]. The release of pro-apoptotic proteins inhibits the negative regulation carried out by inhibitor of apoptosis proteins (IAP) upon procaspases [11]. 

The release of Cyt-c in the cytoplasm activates the formation of the apoptosome, a cytosolic multiprotein complex that is composed of Cyt-c, apoptotic protease activating factor 1 (Apaf-1), and Procaspase-9 (ProCASP9) [12]. The formation of this complex starts from the association of Cyt-c to the cytoplasmic protein structure composed of Apaf-1 monomers. Then, the release of an ATP molecule and assembly of the heptameric apoptosome takes place. After binding and activation of ProC9, the complete apoptosome is formed.

This activation occurs through binding of ProC9 to the caspase recruitment domain (CARD) of the Apaf-1 adapter protein and, once in its active form (CASP9), it must remain bound to the apoptosome to maintain a substantial catalytic activity [13]. Then, the initiator CASP9 cleaves and activates execution caspases, such as Caspase-3 (CASP3) and Caspase-7 (CASP7), through proteolysis that rearranges critical protein loops in the formation of active sites [14,15]. When activated, execution caspases can cleave and activate other execution caspases in a feedback system during the execution phase [16]. See Execution phase for more detail on caspases.

It is also important to note that, in addition to apoptosome, other protein platforms that trigger specific mechanisms not only in the intrinsic, but also in the extrinsic pathway of apoptosis are currently known and they are all related to diverse initiator caspases [17]. Among these structures, here we highlight PIDDosome, ripoptosome, and FADDosome, of which PIDDosome is involved in the intrinsic pathway. Ripoptosome and FADDosome will be further discussed. 

The PIDDosome complex was first described by Tinel and Tschopp [18] as a complex composed by PIDD (p53-induced protein with a death domain) protein, RAIDD (RIP-associated ICH-1/CED-3-homologous protein with a death domain) adaptor, and caspase-2, leading to the activation of the latter. PIDDosome has been suggested to be involved in p53-mediated apoptosis in response to genotoxic stress and DNA damage, but recently it has been associated to other non-apoptotic roles, such as centrosome surveillance during cellular differentiation [19]. 

## 3. Extrinsic or Death Receptor Pathway

The extrinsic pathway of apoptosis starts from external stimuli to the cell. It is triggered by the oligomerization of transmembrane proteins from the superfamily of death receptors or when extracellular concentrations of specific factors reach a particular threshold, leading to the transduction of lethal signals or dependence receptors [20].

During the extrinsic pathway, mitochondria amplify caspase activation, but are not considered essential to this type of cell death [20]. Instead, the extrinsic signaling pathway is triggered when DR are activated by receptor binding or aggregation. Such DR belong to the tumor necrosis factor (TNF) receptor superfamily, characterized by cysteine-rich extracellular domains, as well as cytoplasmic death domains [21]. The most well-known members of this superfamily are Fas, TNFR1, DR3, DR4, DR5, and DR6 [22]. Some of these death receptors, such as Fas and DR5, can be activated by different proteins, such as p53 and MYC. The latter interacts and leads to activation of DR5/TRAIL and Fas death receptors, while the former induces DR transcription, in addition to increasing Fas and DR5 levels in the cytosol by non-transcriptional mechanisms [23,24,25].

There are eight types of death receptors (DR1–DR8), which can be divided in two groups according to the adapter protein [26,27]:

(i) The first group includes the receptors Fas (DR2) TRAILR1 (DR4) and TRAILR2 (DR5), which can be activated by Fas ligand (FasL) and tumor necrosis factor-related apoptosis-inducing ligand (TRAIL), respectively. TRAIL ligands bind to a DR and a TRAIL receptor (TRAIL-R), triggering apoptotic signals and inducing the formation and activation of death-inducing signaling complex (DISC) complex [28]. Once activated, these receptors recruit the death-inducing signaling complex (DISC)—which is composed of FAS-associated via death domain (FADD) and Procaspase-8 (ProC8)—for the transduction of apoptotic signals;
(ii) The second group includes the receptors TNFR1 (DR1), TRAMP (DR3), DR6, and EDAR. These DRs recruit TNF-associated death domain (TRADD) as adapter protein, and bind to the TNF-2,5 receptor-associated factors (TRAF2,5), the receptor-interacting protein kinase (RIP1 or RIPK1), and to cellular inhibitors of apoptosis proteins (cIAP). This forms a signaling complex, called Complex I, for signal transduction of apoptosis and cell survival.

Once DISC is formed, caspase-8 (CASP8) is activated, leading to the activation of other caspases, such as CASP3 [22]. However, the binding of CASP8 to FADD and the further formation of DISC may be prevented if an isoform of one of the DR regulators, called cellular FLICE-like inhibitory protein (cFLIPs), bind to FADD [27]. This is because one of these cFLIP isoforms has a highly similar structure to CASP8, missing only the active catalytic domain. The important regulatory role of cFLIPs is reviewed in Tsuchiya et al. [29].

In addition, FADD-dependent apoptosis and CASP8 may be indirectly induced by TNFR1, as shown in Figure 1. The TNFR1 receptor recruits the TNFR1-associated death-domain protein, which serves as a platform to recruit different signaling molecules: (i) FADD, which functions as a mediator of the activation of apoptosis through the activation of CASP8, and (ii) TRAF2 (in the TRAF2/cIAP complex) and RIP, eventually leading to the activation of NF-κB and JNK/AP-1 (c-Jun NH2-terminal kinase/Jun proto-oncogene, AP-1 transcription factor subunit), which possess anti-apoptotic activity [21]. As previously mentioned, among the different protein platforms in extrinsic pathway, there are ripoptosome and FADDosome.

Ripoptosome was described by Tenev et al. [30]—and independently by Feoktistova et al. [31]—as composed by RIPK1, FADD, and CASP8 in response to loss of XIAP, cIAP1, and cIAP2 activity induced by genotoxic stress. These IAPs can suppress ripoptosome formation, which can also be negatively regulated by FLIP. This complex is able to convert proinflammatory cytokines into death signals and it may lead to cell death through apoptosis and necroptosis due to RIPK1 function in both types of cell death, which can be modulated by IAPs and cFLIP [30,31].

As described by Henry and Martin [32], FADDosome is a pro-inflammatory complex induced by TRAIL-R stimulation and composed by CASP8, FADD, and RIPK1, in which CASP8 presents non-enzymatic functions in this formation, in addition to CASP8 also being able to lead to NF-kB activation. According to Mohr et al. [33], FADDosome is composed not only by these proteins, but also by CASP10 and TRAF2, and it is mainly induced by DNA damage—protein kinase ATR is essential to FADDosome formation through CASP10 upregulation. In the presence of CASP8 and absence of the other factors in this scenario, FADDosome cannot be assembled and CASP8 mediates cFLIPL cleavage generating an alternative structure called FLIPosome, which leads to NF-κB and TNF-α activation [33]. As highlighted by Mouasni and Tourneur [34], FADDosome seems to be important in inflammatory pathways and tumor growth. Considering that FADDosome has been recently described, much is still to be discovered on this complex.

Nevertheless, induction of apoptosis and activation of NF-κB is considered to be inhibitory to each other: The apoptotic process can be interrupted at different points by the NF-κB pathways, being influenced by the accessibility to IAP proteins or the activation of Akt pathways, and an apoptosis process already in progress may block NF-κB signaling pathway through the cleavage of components by caspases [35,36]. Furthermore, Akt activation prevents Cytochrome C release and directly inhibits proapoptotic signals through the phosphorylation and cytoplasmic sequestration of BAD and the O subfamily of the forkhead box transcription factors (FoxOs) [37,38,39]. FoxOs are involved in a series of cellular functions, such as cell survival and tumor suppression pathways, upregulation of pro-apoptotic molecules, such as Bim and Fas-L, and FoxO’s inhibition leads to decreased expression of TRAIL [40,41,42].

## 4. Execution Phase

**Figure 2:**
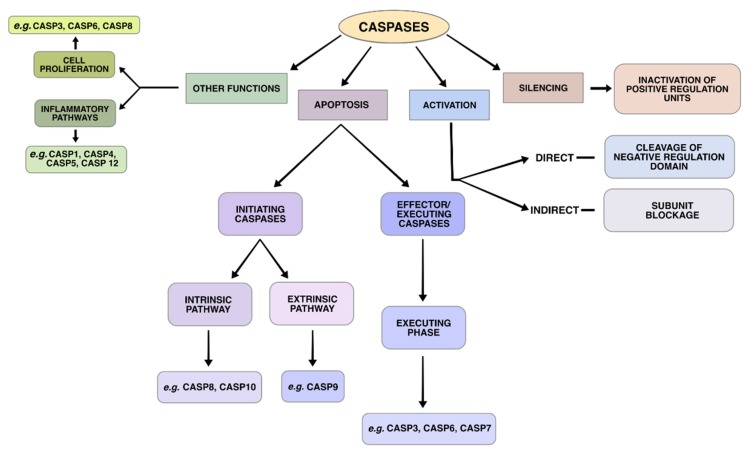
The main roles played by caspases in different biological pathways, including apoptosis.

There are many molecules involved in the apoptotic process, among which caspases are one of the most important, and participate in both of the aforementioned pathways. Caspases (*cysteine-aspartic proteases*) (Figure 2) are endoproteases that hydrolyze peptide bonds in a reaction that depends on catalytic cysteine residues. The above-mentioned series of molecular events lead to the activation of caspases, which then cleave a restricted set of target proteins in the primary sequence, generating either protein inactivation or direct (by cleavage of a negative regulatory domain) or indirect (inactivating a regulatory subunit) activation of proteins [43].

There are many types of caspases (Figure 2), namely Caspases -2, -3, -6, -7, -8, -9, -10 and Caspase-activated DNAse (CAD) [44,45]. These proteins are classified as initiators (e.g., CASP2, CASP8, CASP9, and CASP10) or effectors/executioners (e.g., CASP3, CASP6, and CASP7) according to their role in apoptotic pathways [46]. Initiators activate executioners, which in turn activate other executioners in a feedback loop, affecting essential structural proteins and other enzymes, so that key features of apoptotic cell death occur [16].

Caspase classification entails sequence similarity, substrate selection, and catalytic activity. The determination of such features may be assessed though in vivo and in vitro biological assays. Murine caspase knock-out models have been useful for cellular functions carried out by multiple caspases [47]. In some cases, this has also elucidated other roles, such as CASP3, -8, and -9 deficiency leading to embryonic lethality [48,49,50]. The variety of caspases acting in apoptosis has previously suggested possible redundancies. However, proteomic assays have determined more specialized roles [51,52,53]. It is worth mentioning the unique case of initiator CASP10, which is only found in humans and has had its specific biological roles elucidated mainly by in vitro experiments [54].

The activation of the executioner caspases in the intrinsic or extrinsic pathway is considered to be the beginning of apoptotic execution phase, the final part of this cell death process. Executioner caspases activate cytoplasmic endonucleases that degrade nuclear material and proteases that degrade core and cytoskeletal proteins [2].

Among the executioner caspases stands out the role of CASP3, which activates the nuclease CAD through controlled cleavage of the Inhibitor of CAD (ICAD) [55]. CAD is a nuclease that induces DNA breakage within the nuclei, frequently through the p53 protein signaling [56,57]. Another key substract activated by CASP3 is an actin-binding protein named Gelsolin [58]. CASP3 will cleave gelsolin and the cleaved fragments of the protein will cleave actin filaments [2]. 

This results in cytomorphological changes that include cell shrinkage, chromatin condensation, formation of cytoplasmic blisters and apoptotic bodies, with consequent phagocytosis of bodies by adjacent, neoplastic, or macrophage cells. 

## 5. Genes Involved in the Apoptotic Pathways

Here, we focus on the roles played by nine genes that have essential functions in the intrinsic (*APAF1*, *BCL2,* and *CASP9*), extrinsic (*CASP8*, *FADD,* and *FAS*), both pathways (*TP53* and *MYC*), and in the execution phase (*CASP3*).

## 6. Intrinsic Pathway

### 6.1. APAF1 (Apoptotic Peptidase Activating Factor 1)

The *APAF1* gene (Apoptotic peptidase activating factor 1) encodes the Apaf1 protein, which is activated by Cyt-c and regulated by pro-apoptotic molecules (such as Bax and Bid) and anti-apoptotic molecules (such as Bcl-X and Bcl-2). As part of the apoptosome, it is responsible for the activation of CASP9, which leads to CASP3-dependent cell death [59]. 

Prior to apoptosome formation, Apaf1 is normally present in the cytoplasm in the form of inactive monomers bound to ATP molecules and undergoes conformational changes for binding to Cyt-c [13]. Because it is the intermediate between Cyt-c and ProC9, Apaf1 is considered the main component of apoptosome [12].

Recently, the role of Apaf1 as a major apoptosis regulator was demonstrated in a human embryonic stem cell assay, which concluded that low levels of *APAF1* expression were possibly determinant for inhibiting cell death in neuronal development [60]. This is further reinforced by the identification of deleterious variants in the *APAF1* gene in human fetuses with neural tube defects, showing the significant impact that mutations in apoptotic genes may have in human development [61]. 

### 6.2. BCL2 (B-Cell Lymphoma Protein 2, Apoptosis Regulator)

The *BCL2* (B-cell lymphoma protein 2, apoptosis regulator) gene encodes for its homonymous protein, is part of the Bcl-2 (B-cell lymphoma protein 2) family, and acts as pro-survival [7]. This family has at least nine members described, including antiapoptotic members (BCL-2, BCL-X_L_, MCL-1, BCL-w, BFL-1/A1), pro-apoptotic members (BCL-B, BAX, BAK, and BOK), and four similar proteins (BCL2L13, BCL2L14, BCL2L15, and BID) [62,63]. BCL-B has four BCL-2 Homologous domains (BH), from which pro-apoptotic BH3-only proteins (BAD, BCL-X_S_, BLK, and BLD) are derived [64]. The balance between pro-apoptotic and anti-apoptotic Bcl-2 proteins influences the sensitivity of cells to apoptotic stimuli [65]. For example, the increase of mitochondrial cAMP leads to the cAMP-dependent protein kinase (PKA)-mediated phosphorylation of BAD, preventing its heterodimerize with Bcl-2 and Bcl-X, thus causing cell survival [66,67]. However, when dephosphorylated, BAD and Bcl-2 are translocated to the mitochondria, and their heterodimerization leads to the release of cytochrome c in the cytoplasm, hence leading to apoptosis [68,69,70].

Considering apoptosis evasion is one of the hallmarks of cancer, it is expected that apoptotic genes are found translocated or mutated in the tumor environment [71]. For the *BCL2* gene, this context was crucial to its discovery. First described in follicular B-cell lymphoma, the *BCL2* gene is located in the 18q21.3 region and can be linked to a locus in a translocation [72,73] that increases transcription of its protein and causes gain of function [74,75]. 

*BCL2* has been well-established as a therapeutic target due to its important roles in apoptosis, and has a number of pharmaceutical initiatives in cancer treatment [76]. Interestingly, a new acquired mutation in *BLC2* was recently described inhibiting drug efficacy in chronic lymphocytic leukemia. This single mutation caused a 180-fold reduction in venetoclax binding affinity to BCL2, demonstrating how decisive apoptotic genes are in the context of human disease [77]. In addition, the interactions of Bcl-2 with cellular pathways, such as Akt and RAS, is also important since it can be a mechanism for evasion of apoptosis and chemoresistance in cancer cells [78,79,80].

### 6.3. CASP9 (Caspase 9)

The *CASP9* gene encodes the homonymous protein that acts as the initiating caspase in the intrinsic apoptotic pathway. Its inactive form, ProC9, is composed of three distinct regions: An N-terminal caspase recruitment domain (CARD), a large catalytic subunit, and a small catalytic subunit. Conformational changes lead to the formation of the apoptosome, which seems to serve as a platform for increased concentration and subsequent activation of CASP9 [13]. The active form of this protein must remain attached to the apoptosome to maintain its catalytic activity [81].

The relative concentrations of Apaf-1 and ProC9 appear to influence the proportion of CASP9 homodimers and heterodimers formed in apoptosomes; that is, apoptosomes seem to function as a mediator for the formation of homodimers (induced by Pro-C9 proximity) and heterodimers (resulting from the interaction between the small subunit of ProC9 and the NOD domain of Apaf-1), culminating in the activation of caspases such as CASP3, CASP6, and CASP7 [82,83].

It was demonstrated that high inflammatory responses are associated to increased CASP9 activity and this process may be involved in the progression of inflammatory conditions, such as periodontitis and acute liver disease [84,85]. Furthermore, variants of *CASP9* have been associated with cancer [86].

## 7. Extrinsic Pathway

### 7.1. CASP8 (Caspase 8)

The *CASP8* gene encodes a homonymous protein, which is synthesized as a zymogen and is the first activated caspase in the extrinsic pathway, which occurs after the formation of the DISC complex [87,88,89].

Active CASP8 concentration at this point determines which apoptotic pathway to follow: If CASP8 protein levels are high, it directly cleaves CASP3, which then performs its function; if levels are low, it truncates BID (tBID), which releases Cyt-c from mitochondria, leading to the activation of CASP9, which in turn cleaves CASP3 [90]. Thus, the main function of CASP8 seems to be the activation of executing procaspases, especially ProCASP3. Recently, increased CASP8 plasma concentration was shown to be a candidate biomarker for diabetes mellitus (DM) because it enhances β-cell apoptosis, leading to both circadian rhythm disruption and DM incidence [91]. This discovery reveals that apoptotic activity is related to sleep duration and that CASP8 levels can indicate incidence of an endocrine disease years before onset.

Additionally, *CASP8* alterations have also been observed in disease contexts in roles aside from its apoptotic functions. For instance, non-apoptotic functions of CASP8, such as those involved in immune response, are critical in the development of very early onset inflammatory bowel disease through interactions with the immune system [92,93].

### 7.2. FADD (FAS-Associated Via Death Domain)

The *FADD* gene (FAS-associated via death domain), also known as *MORT1*, encodes the FADD protein, which acts as an adapter in the extrinsic pathway. It was discovered and associated with interaction with the Fas receptor by Chinnaiyan et al. [94].

FADD is considered the main adapter for the transmission of DR-mediated apoptotic signals, since the interaction between this adapter and the majority of death receptors has already been described [95]. FADD protein consists of two domains that interact with other domains: Death domain (DD) and death effector domain (DED) [27]. DD/DD interactions lead to recruitment of FADD, initiating the formation of DISC, while DED/DED interactions recruit CASP8 or CASP10 [96].

In addition to its functions in apoptosis, FADD can also induce cell survival, cell cycle progression, and cell proliferation, depending on its phosphorylation and cellular localization [97]. Disrupted FADD phosphorylation has been inversely correlated with tumor aggressiveness in T-cell lymphoblastic lymphomas [98]. Moreover, decreased FADD is associated with cognitive impairment and clinical dementia in the elderly [99]. In addition, a recent review has explored the roles of FADD in the context of cancer and inflammation, highlighting the impact of translational and post-translational modifications in its multiple functions [34].

### 7.3. FAS (FAS Cell Surface Death Receptor)

The *FAS* gene (FAS cell surface death receptor), also known as *CD95* and *APO-1*, encodes FAS (or Fas), a type I transmembrane protein located on the cell surface. It is considered a DR due to its main role of inducing cell death when binding to FasL [96]. This binding induces the trimerization of Fas in the cell membrane, which leads to its activation and recruitment of FADD [90].

In addition to its well-defined function in apoptosis, Fas also appears to have non-apoptotic functions: It is involved in the activation of RIPK1, resulting in cellular necrosis; and it may also activate other signaling pathways, resulting in cell differentiation or proliferation [95].

Polymorphisms in *FAS* gene may decrease the apoptotic potential of the receptor, and have been associated with several illnesses, including cancer and musculoskeletal degenerative disorders [100,101]. Furthermore, there are studies demonstrating the association of *FAS* polymorphisms with the persistence of HTLV infections and its clinical manifestations, such as Adult T-Cell Leukemia (ATL) and HTLV-1-associated myelopathy (HAM) [102,103,104].

## 8. Intrinsic and Extrinsic Pathways

### 8.1. TP53 (Tumor Protein p53)

The *TP53* tumor suppressor gene (Tumor protein p53) encodes the p53 protein, a transcription factor that has different functions involving cell and genomic stability, senescence, and cell death. The p53 protein targets more than 900 genes, affecting not only apoptosis, but also metabolism, proliferation, and immune response genes [105]. 

It has also been demonstrated to induce apoptosis independently of transcription in both pathways. This protein activates the extrinsic pathway by inducing *FAS* and promoting apoptosis through an element located in intron 1, which binds to *FAS* promoter regions and reaches maximum transactivation [106]. Its mechanisms through DNA damage may also be implicated in DR5 activation and consequent activation of CASP8 and Poly ADP-ribose polymerase (PARP), with this relationship being a potential therapeutic target in cancer treatment [107]. Recently, a component named Ziyuglycoside I (Ziyu I) was reported to be capable of inducing mitochondrial dependent apoptosis in human WERI-Rb-1 Retinoblastoma cells due to increased expression of p53 [108].

In addition, p53 positively regulates the transcription of the intrinsic pathway factors Noxa, PUMA, and BID that induce apoptosis when highly expressed [109]. Moreover, p53 is necessary for the formation of the apoptosome, by inducing the Apaf1 promoter. It also activates CASP8 and CASP6 in response to cell stress and DNA damage, respectively. P53 localization to the mitochondria also promotes apoptosis by triggering the BAX channel on the mitochondrial surface, responsible for releasing Cyt-c, which activates CASP9 and gives rise to the initiation of apoptosis [110]. 

### 8.2. MYC (MYC Proto-Oncogene, bHLH Transcription Factor)

The *MYC* gene (bHLH transcription factor), also known as *c-Myc*, acts on the transcriptional regulation of several processes, including cell progression and apoptosis [111] in conjunction with partner protein Max [112,113]. The dual roles played by *MYC* for being an oncogene and a pro-apoptotic protein are determined by expression levels. For this gene to take part in cell death, the threshold of expression must be much higher than for cell cycle induction, which involves a highly regulated mechanism [114]. 

Regarding apoptosis, besides its involvement in the extrinsic pathway, MYC is known to drive cell death through p53-mediated apoptosis. MYC activation leads to increased expression of tumor suppressor p14ARF, which in turn blocks p53 inhibitor hMDM2 and ultimately increases p53 expression and the mitochondrial pathway [115]. However, p53-independent MYC-induced apoptosis has been described in certain cell types [116,117]. The main mediator of MYC-induced cell death, however, is reported to be one of its BH3-only targets (BIM) also in a p53-independent mechanism [118]. 

It seems counter-intuitive that a gene capable of inducing apoptosis at high expression levels may also be related to increased tumorigenesis. However, mechanisms through which MYC triggers apoptosis have been proved to also lead to genomic instability, which explains this paradox [119]. It is important to highlight that MYC is an essential global transcription regulator, capable of controlling up to 15% of genes in the human genome [120,121]. 

## 9. Execution Phase

### CASP3 (Caspase 3)

The *CASP3* gene encodes a homonymous protein that is considered to be the most important effector caspase. During the execution phase of apoptosis, CASP3 activates the CAD endonuclease (Caspase-activated DNAse) by cleaving it from its inhibitor (ICAD). After the release of CAD, it degrades chromosomal DNA, causes chromatin condensation, and induces cytoskeletal reorganization and cell disintegration in apoptotic bodies [2]. It has been suggested that CASP3 has other roles and some that overlap with those of CASP6 and CASP7 [46].

Despite being dependent on chain cleavage and, consequently, initiator proteins, the apoptotic functions of CASP3 have been shown to work in the absence of such activator molecules. This protease may be constitutively activated in the presence of mutation V266E, indicating a possible therapeutic strategy for apoptosis induction [122]. Caspase 3 deregulation studies demonstrate that, in colorectal cancer, CASP3 acts to promote tumor invasiveness and metastasis after exposure to radiation and chemical carcinogens [123], hence it is suggested that targeting *CASP3* may increase tumor susceptibility to cancer treatment and inhibit metastasis [124].

## 10. Mitochondria and Epigenetic Regulation

Mitochondria are cytoplasmic organelles responsible for the generation of cellular energy (ATP) through the process of oxidative phosphorylation (OXPHOS). They have several other functions that are also essential for normal cell functioning, such as control of calcium levels, lipid homeostasis, metabolic cell signaling, and cell death [125]

During OXPHOS, mitochondria generate reactive oxygen species (ROS) through the electron transport chain (ETC). Despite being a natural part of the process, an accumulation of ROS due to a shift in equilibrium causes damage to the mitochondrial DNA (mtDNA) [126]. When a cell is no longer functioning normally, it initiates regulated cell death, most commonly apoptosis. Mitochondria not only have an essential role in intrinsic apoptosis, but also in providing energy for the complete apoptotic process [127].

In the intrinsic pathway, pro-apoptotic proteins from the Bcl-2 family translocate to the mitochondrial outer membrane and mediate its permeabilization, inducing the release of Cyt-c and IAP inhibitors. This permeabilization is also controlled by certain mitochondrial dynamics, which involve regulation of the organelle’s fission (mediated by Dynamin-related protein 1) [127], fusion of the outer membrane (role played by Optic atrophy 1) [128], lipid content (such as ceramides) [129], and interaction with the endoplasmic reticulum (ER) [130]. 

In the absence of caspase involvement, mitochondria can undergo apoptosis upon Bax/Bak-induced MOMP, by triggering the release of mtDNA, resulting in IFN expression and a pro-inflammatory type of cell death. Furthermore, the simultaneous activation of caspases inhibits the aforementioned process, meaning mitochondria have a pivotal role in deciding on which pathway it will carry out cell death, a possibly inflammatory or immunologically silent process [131,132]. The extrinsic pathway requires the mitochondria downstream from the activation of death receptor proteins in certain cell types, which interact with this organelle upon the CASP8-mediated activation of Bid, a pro-apoptotic Bcl-2 member [133,134].

Alternatively, apoptosis may be triggered by high levels of calcium ions in the ER. Apoptotic stimuli cause the release of intracellular calcium from the ER and thereby an overload in mitochondria, leading to increased membrane permeability and subsequent flow of pro-apoptotic molecules into the cytoplasm [135].

Considering the number of interacting molecules involved in mitochondrial-mediated apoptosis, it is expected that mutations in mtDNA genes may lead to differences in cell death processes. Additionally, a level of complexity is added once we acknowledge the effects of heteroplasmic variations in mitochondrial genomes. Seeing that the number of mitochondria in a cell may vary widely depending on cell type, proportions of a given mutation are also subject to such fluctuations [136]. This state in which different genotypes of a mutation coexist in the mitochondria of the same cell is called heteroplasmy, while the equality state of the genotypes is called homoplasmy. Studies have shown that different levels of heteroplasmy may have a significant importance in mtDNA and nuclear gene expression, including genes involved directly and indirectly in apoptosis and tumorigenesis [137,138].

Interestingly, much like the nuclear genome (nDNA), mtDNA is also controlled by epigenetic mechanisms. The epigenetic events related to mitochondria are known as *mitoepigenetics* and are carried out by mtDNA methylation and non-coding RNAs (ncRNAs) [139]. 

While nDNA methylation is a well-established process, mtDNA methylation is still controversial and poorly understood. However, some studies have demonstrated the presence of nuclei-encoded DNA-methytransferases (DNMTs) inside human mitochondria [140,141]. These enzymes cause DNA modifications by adding a methyl group at the 5′ position of cytosine bases of both nuclear and mitochondrial genomes, resulting in transcriptional regulation [139,142]. 

Actually, mtDNA methylation levels were related not only to gene expression regulation, but also to mtDNA copy number. Several diseases have been studied from a mitoepigenetic point of view and the evidences suggest that abnormal mtDNA methylation is associated to pathological conditions, particularly to neurodegeneration and aging [143]. Increased DNMTs activity in mitochondria was related to adaption to oxidative stress, suggesting that mtDNA methylation is an apoptotic-related mechanism. In this context, higher levels of mtDNA methylation were found in neuronal mitochondria from patients with amyotrophic lateral sclerosis, indicating that motor neurons apoptosis is driven by DNMT upregulation [139]. 

Non-coding RNAs are a broad class of regulatory RNAs whose function is not to serve as templates for protein production. Most ncRNAs do not have their roles fully understood, but they were described acting at transcriptional, posttranscriptional, and posttranslational levels, meaning that they can interact with DNA, other classes of RNAs, and proteins. They are classified into two major types according to their length: Long ncRNAs (≥200 nt) and small ncRNAs (<200 nt), which include microRNAs (miRNAs; 17–23 nt), short interfering RNAs (siRNAs; 20–30 nt), and piwi-interacting RNAs (piRNAs; 27–30 nt) [144,145]. 

Our knowledge about ncRNAs and mitochondria is still very poor, but the evidence suggests that they may have major roles in retrograde and anterograde signals in cellular environment. It is important to note that there are two different contexts inside mitochondria regarding ncRNAs: There are ncRNAs produced by the nuclear genome, which can then be found inside the organelle, and those that are encoded by the mitochondrial genome, which can be found inside and outside the organelle. In general, the first scenario is related to anterograde signals and the latter is associated to the retrograde ones [143,146,147]. 

So far, there are some long ncRNAs and miRNAs that were described in association with mitochondria in both contexts. Considering the first context, the nuclei-encoded long ncRNA MEG3 was found in mitochondria enhancing apoptosis by reducing the expression of Bcl-2 and ProC9 proteins and promoting the release of Cyt-c to the cytoplasm [148]. Additionally, 15 nuclear-encoded miRNAs were identified in mitochondria of mice, and the functional enrichment suggested their role in cell death (including apoptosis) and cell division [149]. 

Regarding the second context, studies describing the mtDNA encoding ncRNAs are very sparse. To date, the most studied ncRNAs in this context are ASncmtRNA-1 and ASncmtRNA-2, two types of antisense long ncRNAs derived from mtDNA. They were found inducing apoptosis in several human and mouse tumor cell lines and involved in aging and replicative senescence in normal human cells [150,151]. In 2011, the mitochondrial transcriptome allowed the identification of 31 mitochondrial-encoded small ncRNAs, which presented a large and dynamic range of expression within different cell types, but that still have their biological roles unknown [152]. 

Overall, very little attention has been devoted to mitochondrial epigenetics, but it is very clear that mitochondria play a critical role in nearly all aspects of apoptosis, ranging from pro-apoptotic protein release, to morphological traits, epigenetic mechanisms, and gene expression regulation, demonstrating the need for a careful analysis of its many layers of complexity. 

### miRNAs in Apoptosis

MicroRNAs (miRNAs) are small non-coding RNAs that regulate gene expression and many cell processes, including apoptosis. Indeed, it appears that miRNAs have a role in apoptotic pathways mainly in two forms: (i) Acting as anti-apoptotic molecules by targeting pro-apoptotic messenger RNAs (mRNAs) or positive regulators of pro-apoptotic mRNAs, (ii) as pro-apoptotic miRNAs by targeting anti-apoptotic mRNAs or their positive regulators [153]. These roles may be performed upon both the intrinsic and extrinsic machinery of the apoptosis pathway. In fact, many studies have indicated that miRNAs may directly or indirectly influence cancer treatment by regulation of apoptotic cell death through intrinsic or extrinsic pathways. 

For instance, miR-491 targets BCL-XL, decreasing cell viability by inducing apoptosis and generating tumor suppression in DLD1 colon cancer cells [128]. miR-133a targets both BCL-XL and MCL-1 and, when upregulated, it has been shown to induce apoptosis and suppress tumorigenesis in osteosarcoma cell lines [154]. Similarly, BCL-XL and epidermal growth factor receptor (EGFR), both tumor suppressors, are also targeted by has-miR-608 [155]. Furthermore, miR-365 acts directly by targeting adaptor protein Src Homology 2 Domain Containing 1 (SHC1) and BAX, promoting tumor cell resistance to gemcitabine and consequent cell proliferation [156]. 

Moreover, many miRNAs have been reported to influence Bcl-2 expression in the intrinsic pathway. miR-15 and miR-16 negatively regulate Bcl-2; in fact, transfection of both miRNAs in cells lines that express high levels of Bcl-2 expression reduced these levels and resulted in the activation of intrinsic apoptosis pathway [157]. Pro-apoptotic activity of miRNA in response to 5-fluorouracil has been reported in gastric cancer cell lines with down-regulation of the miR-204, which results in ectopic expression of Bcl-2; on the other hand, ectopic expression of miR-204 leads to down-regulation of Bcl-2 and determines colony formation and migration [158]. 

Similarly, p21-activated kinase 2 (PAK2) and Cyclin D3 (CCND3) were identified as direct targets of miR-4779, a miRNA that inhibits cancer cell growth by inducing apoptosis and cell cycle arrest. Moreover, miR-4779 was downregulated in 9 out of 10 colon cancer tissues, while the expression of PAK2 and CCND3 was significantly higher [159]. miR-378 suppresses intrinsic apoptosis initiation directly through targeting the initiator CASP9; miR-378/378* knockout mice cannot maintain normal muscle weight and display impaired autophagy, accumulation of abnormal mitochondria, and excessive apoptosis in skeletal muscle [160]. The proapoptotic protein BIM, involved in controlling the mitochondrial apoptotic machinery, is regulated by miR-20, miR-92, and miR-302. Therefore, these miRNAs are essential for maintenance of cell survival in stem cells that are primed not only for differentiation but also for cell death [161].

The examples above were mainly focused on intrinsic pathway. However, extrinsic pathway is also regulated by several miRNAs. For instance, overexpression of miR-221 and miR-222 leads to cell sensitivity to TRAIL in resistant tumor cells and induces apoptotic cell death [162]. It has been observed that such miRNAs negatively modulate the expression of proto-oncogenes Kit and p27kipl and reduce resistance to tamoxifen by inducing apoptosis in breast cancer cell lines [163]. In pancreatic cancer resistant to gemcitabine-induced apoptosis, downregulation of FasL and upregulation of its direct regulator miR-21 have been observed [164]. The same has been reported for another FasL regulator, mir-590, in acute myeloid leukemia (AML), promoting cell survival [165]. 

In osteosarcoma cells, miR-20a has been shown to inhibit Fas expression, promoting an increase of tumor cell survival and enhancing its metastatic capacity [166]. Upregulation of miR-25 also seems to protect tumor cells against TRAIL-induced apoptosis in cholangiocarcinoma [167,168]. Several other miRNAs target proteins in the TRAIL pathway. For instance, it has been predicted that miR-182 and miR-96 target *FADD* and *CASP3*, while miR-145 and miR-216 target the receptors of *TRAIL*, *DR4,* and *DR5* [23]. As for other extrinsic mechanisms, miR-K10a is an oncomiR that increases cell survival and downregulates the expression of apoptotic tumor necrosis factor receptor superfamily member 12A (TWEAK) receptor [169]. In addition, miR-128a targets *FADD* and it has been observed that transfection of mimic miR-128a has determined Fas resistance by downregulating *FADD*, while miR-128a antagomir has induced Fas-mediated apoptosis by active FADD expression [170].

It is now well-established that cell death metabolic regulation is a complex and sophisticated mechanism with multiple checkpoints to regulate cell fate in response to multiple stimulus and micro-environment, which can lead to apoptosis, homeostasis, or cell immortality. In this context, these studies highlight the importance of miRNA regulation of different genes involved in metabolic stress and the whole process of apoptosis, as well as miRNA ability to reset the threshold of cell death and attempt to regenerate homeostasis. On the other hand, depending on the target genes, miRNAs can act as anti-apoptotic and generate best conditions to epithelial mesenchymal transition, cell invasion capacity, and subversion of cell cycle arrest. In summary, miRNAs represent a great research field regarding novel therapeutic strategies to management and prevention of various diseases, such as cancer, based on their role in apoptotic pathways.

## 11. Conclusions

Apoptosis is a regulated cell death mechanism of great importance both in the normal development of the body and in the removal of defective cells. Deregulation of this process can lead to the development of different diseases and it can also influence the treatment of some of these diseases. Therefore, it is necessary to understand the mechanisms by which apoptosis occurs and interacts with other biological systems. By doing so, it is possible to discover biomarkers that may affect the clinical progression of many diseases. To assist in this scenario, here we discussed the apoptotic mechanisms and the major cellular and genetic players involved in them.

As a future perspective, it is important to acknowledge the potential interference of other epigenetic agents, besides miRNA and DNA methylation, that have not yet been described in the context of apoptosis and mitochondrial functions. In addition, it would be interesting to investigate variants in genes involved in apoptotic pathways through a population genetics point of view, given that they may have a differential distribution across worldwide populations and that this could be relevant in the development of various diseases. In summary, in the era of precision medicine, the interactive nature of the many molecules involved in apoptosis should be regarded as a new field for disease management.
